# Successful treatment of perineal necrotising fasciitis and associated pubic bone osteomyelitis with the vacuum assisted closure system

**DOI:** 10.1186/1477-7819-6-67

**Published:** 2008-06-24

**Authors:** Susim Kumar, Mark E O'Donnell, Khalid Khan, Gillian Dunne, P Declan Carey, Jack Lee

**Affiliations:** 1 Department of General Surgery, Belfast City Hospital, Lisburn Road, Belfast BT9 7AB, Northern Ireland, Uk

## Abstract

**Background:**

Acute necrotising fasciitis is a life-threatening condition, which requires urgent surgical intervention. Surgical debridement is invariably associated with large areas of tissue loss.

**Case presentation:**

We present a 58-year old woman with a past history of cervical carcinoma who presented with necrotising fasciitis of the perineum and upper thighs with associated pubic bone osteomyelitis. Following extensive debridement, a Vacuum Assisted Closure (VAC) system was applied to the large residual defect to facilitate skin graft application and optimise wound healing.

**Conclusion:**

This case demonstrates the successful management of a complex and potentially lethal wound of the perineum with debridement, skin grafting and the VAC system.

## Background

Necrotising fasciitis (NF) is a devastating soft tissue infection characterised by widespread necrosis of the fascia and subcutaneous tissue. We describe a 58-year old woman who presented with NF of the perineum and thighs which were treated successfully with surgical debridement, broad-spectrum antibiotics, and skin grafting. We emphasise the advantageous use of the vacuum assisted closure (VAC) device which successfully expedited wound healing.

## Case presentation

A 58-year old woman presented to the gynaecology out-patient department with a 1-day history of increasing bilateral hip and suprapubic pain. She had a past history of carcinoma of the cervix 12-years prior to this admission, which was treated with a total abdominal hysterectomy, bilateral salpingo-oophorectomy with adjuvant radio- and chemotherapy. She developed a colo-vaginal fistula and bilateral ureteric obstruction 2-years ago due to complex pelvic sepsis, which was managed by fashioning a defunctioning loop colostomy and an ileal-conduit urostomy. However, the patient was otherwise well with no other significant medical problems.

On examination, the patient was dehydrated and pyrexic (38.6°C). Her blood pressure was 101/56 mmHg and pulse rate was 92/min. Abdominal examination revealed deep suprapubic tenderness with erythema of the perineum and inner thighs. Bowel sounds were normal. Initial haematological investigations demonstrated a haemoglobin level of 12.8 g/dl, white cell count of 19.1 × 10^9^/litre, erythrocyte sedimentation rate of 109 mm/hour and a C-reactive protein of 357 mg/L. She was treated conservatively with analgesia, fluid resuscitation and intravenous antibiotics (benzyl-penicillin-2.4 g, clindamycin-900 mg, ciprofloxacin-400 mg) which were administered 3-times per day. On the second day post-admission, her condition deteriorated significantly and she was transferred to the high dependency unit (HDU) with septicaemic shock with a pulse rate of 110/min, blood pressure of 70/43 mmHg, and an oxygen saturation of 94% on 2 litres/min of oxygen therapy.

**Figure 1:**
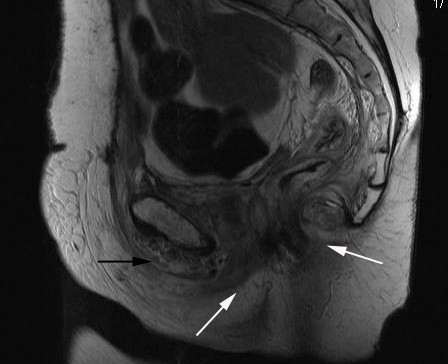
T1-weighted MRI of the pelvis (sagittal view) exhibiting gross oedema of the urethra, vagina, and rectum (white arrows) with an air-fluid level within pubic symphsis extending into peritoneal retropubic space superior to the urethra (black arrow).

An urgent magnetic resonance imaging (MRI) scan of the pelvis revealed extensive oedema of the urethra, vagina and rectum with fluid collections within the proximal thigh adductors bilaterally which contained an air/fluid level within the pubic symphysis with extension into the retropubic space and superior to the urethra (Figure 1). Subsequent surgical assessment identified extensive perineal and inner thigh cellulitis suspicious of necrotising fascititis. She underwent emergency debridement of the necrotic skin and subcutaneous tissues with drainage of pus. Post-operatively, she was transferred to the intensive care unit for inotropic support in the form of intravenous noradrenaline.

Histopathological assessment of a 20 cm × 7.5 cm ellipse of skin showed necrosis of the skin, dermis and subcutaneous fat with evidence of focal inflammation and pus within all tissue layers. Gram staining confirmed gram-positive cocci extending from the surface of the skin into the deep fatty tissue and arranged in chains, suggestive of streptococcal necrotising fasciitis. Cultures of swabs taken from the perineal wound isolated viridans group streptococci, coagulase negative staphylococci, enterococci and mixed anaerobes. The patient underwent repeated exploration and debridement of the wound in theatre under general anaesthesia on days five, seven, nine, twelve and fourteen post-admission.

**Figure 2:**
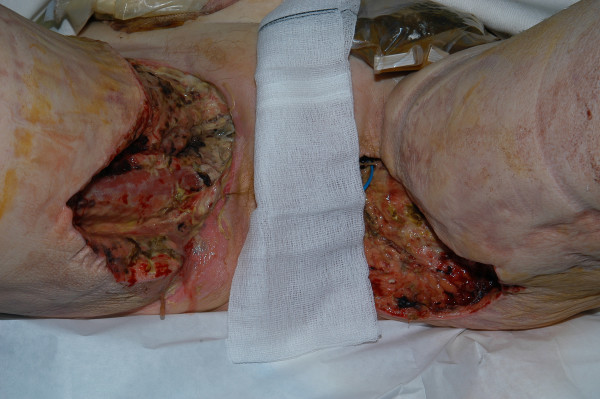
Photograph showing both groin wounds (day-12) with patchy areas of necrotic tissue and slough. The adjacent skin appears healthy with the formation of clean granulation tissue at the wound margins.

**Figure 3:**
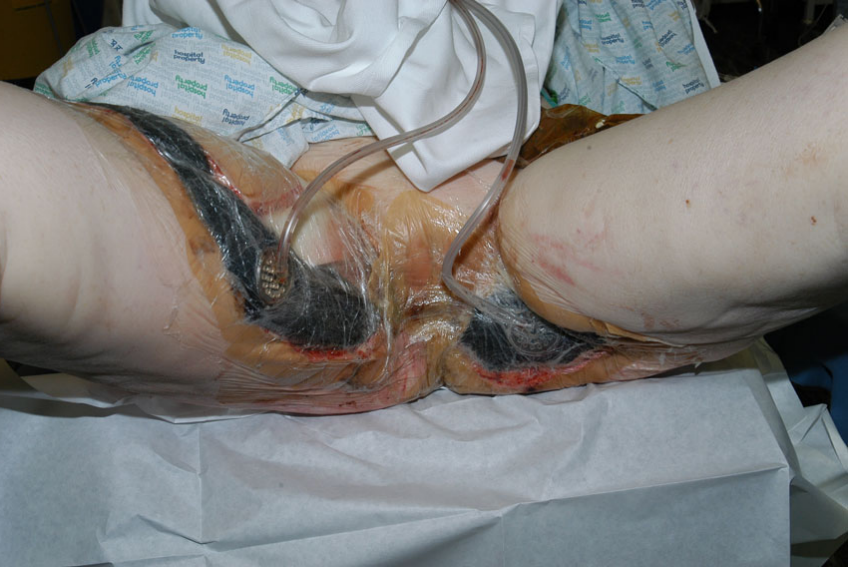
Photograph showing both groin wounds and perineum (day-20) with VAC system in place. The wounds already appeared smaller with healthy adjacent skin.

The wound was suitable for VAC dressing (VAC Therapy™, KCI, Oxfordshire, United Kingdom) from day-9 after initial debridement (Figure 2). Owing to the considerable pain and the position of the wounds, the VAC system had to be reapplied in theatre 3-times a week initially, and then reduced to twice weekly. Sequential wound assessment demonstrated marked improvements with visible granulation tissue following the application of the VAC system set at 125 mmHg continuous topical negative pressure (Figure 3). Skin grafts obtained from the antero-lateral aspect of the left thigh were applied to the perineal and thigh wounds on day-20. The VAC system was then applied over the skin graft at a lower topical negative pressure of 50 mmHg.

**Figure 4:**
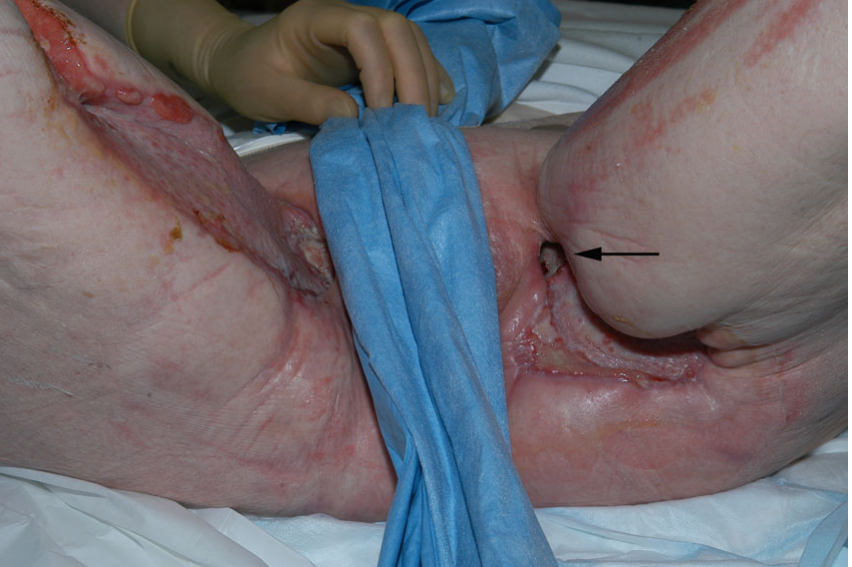
Photograph showing both groin wounds (day-47) healing satisfactorily with evidence of a sinus in the upper part of the left groin wound (black arrow). Adjacent areas appear healthy.

On day-36, a repeat MRI was performed to investigate persistent bilateral groin sinuses. This demonstrated extensive oedema of all the muscle groups around the pelvis, most marked in the region of the obturators and the adductor muscles in the proximal thigh and bone marrow oedema in the symphysis pubis suggestive of osteomyelitis. Biopsy of the symphysis pubis corroborated the presence of osteomyelitis with collections of acute inflammatory cells and some reactive debris in the marrow space in combination with viable and necrotic bone. A gram stain showed some gram-positive organisms similar to those seen on the original biopsy, suggesting a residual nidus of infection in necrotic bone. The patient remained on long-term antibiotic therapy with continued application of the VAC dressing system as further surgical intervention was deemed inappropriate (Figure 4). VAC therapy was discontinued on day-51. The patient was discharged 3-months post-admission with both groin wounds fully healed. She remains well 16-months later with no further signs of soft tissue sepsis or osteomyelitis.

## Discussion

Necrotising fasciitis is a rare, life-threatening soft tissue infection, associated with rapidly progressive inflammation and necrosis of subcutaneous fascial tissues, with or without involvement of underlying muscle [1,2]. Poromanski and Andriessen (2004) reported an incidence in adults of 0.40 cases per 100,000 population [3]. Risk factors include trauma, wound infections, decubitus ulcers, alcoholism, carcinoma, diabetes, peripheral vascular disease, smoking and intravenous drug abuse [1,2,4]. Haematogenous seeding of bacteria to the fascia may be another causative factor of NF and bears significant relevance to our case, as MRI scans of the pelvis and biopsy of the symphysis pubis highlighted the presence of osteomyelitis of the pubic bone, suggesting the possibility of it being a cause rather than a complication or an association of NF [2].

Necrotising fasciitis affects the extremities more frequently than central areas and is classified according to speed of onset and aggressiveness ranging from type 1, a slow evolving process initiated by polymicrobial infection associated with less fulminant systemic complications; to the more aggressive type-2 which is associated with multi-organ failure [2]. Patients may present with high fever, tachycardia and erythematous skin in the early stages. This may progress to more extensive skin involvement, hypoaesthesia, fluctuance and induration [2]. Further deterioration results in haemorrhagic bullae, dermal necrosis, gangrene combined with systemic complications such as hyperpyrexia and septic shock [4]. Mortality rates may reach up to 30% with a higher prevalence exhibited at the extremes of age [2].

Gross fascial necrosis detected at the time of surgical intercession is the gold standard for identifying NF. The 'finger test', which can be performed at the bedside, is based on the discovery of underlying fascial dehiscence. It involves blunt dissection with a probe or digit down to the deep fascia, through an iatrogenic or spontaneous wound. The diagnosis of NF is established if there is effortless dissection of subcutaneous tissue from the deep fascia [4].

Gram-positive group A streptococcus, haemolytic streptococci and staphylococcus aureus; gram-negative enterobacteriaceae, escherichia coli, klebsiella spp and proteus spp; anaerobes including peptostreptococcus, clostridia and bacteroides; fungi such as candida, and acid fast bacteria have all been implicated in the pathogenesis of necrotising fasciitis [2]. However, wound cultures are often sterile due to prior administration of antibiotics. Bacteriological culture from our case grew a mixture of microbes including streptococci, staphylococci, enterococci and anaerobes. It is important for early clinical assessment to detect subtle changes associated with fascial necrosis, suggestive of NF. Plain radiography can detect subcutaneous gas while computerised tomography and magnetic resonance imaging are more sensitive to diagnose NF and to differentiate other causes of soft tissue infection, such as abscesses [2].

Intravenous antibiotic administration must not be delayed if necrotising fasciitis is suspected clinically. The antibiotic must have broad-spectrum properties and be effective against gram-positive organisms, gram-negative rods and anaerobes. Carboxypenicillin, carbapenam, clindamycin and metronidazole have been used successfully in various combinations to treat NF. Intensive nutritional supplementation, haemodynamic and analgesic support are all important for improving survival. Some studies have shown a reduction in the morbidity and mortality with the use of adjunctive therapies such as intravenous immunoglobin and hyperbaric oxygen [2].

However, aggressive early surgery is the single most important influence on the survival rates of patients affected with NF. Patients need to undergo immediate and extensive resection of all devitalised and necrotic tissue. Wong *et al *(2003) reported a mortality rate of 6% for surgery conducted within 24-hours compared to a rate of 24% if performed between 24 and 48 hours [5]. Our patient required 6 wound debridements within 2-weeks of admission. Further reconstruction with skin grafting and flaps combined with defunctioning procedures are indicated for the prevention of wound contamination in abdominal and perineal cases of NF.

Vacuum-assisted wound closure (VAC) requires the application of an adhesive sterile seal around the wound combined with a continuous or intermittent negative external pressure. This technique involves the application of an open-cell foam onto the wound followed by the application of an adhesive cover to seal the wound from external contamination to facilitate the application of controlled sub-atmospheric pressure (Figure 3) [6]. Circulation is enhanced 4-fold, with increased rates of granulation tissue formation, lowered bacterial counts and enhanced flap survival [4,6]. Serial debridements combined with time-consuming painful daily dressings are avoided. The VAC system removes excess wound exudate and decreases oedema [7]. It facilitates early ambulation combined with a reduction in hospital stay, morbidity and mortality rates. Phelps *et al *(2006) demonstrated the effectiveness of the VAC system compared to the traditional wet-to-dry dressings with a time for wound healing advantage of approximately 3-weeks [4].

## Conclusion

NF associated with underlying osteomyelitis is extremely uncommon with only one previous case report of scalp necrotising fasciitis with aspergillus osteomyelitis of the skull [8]. Although perineal NF with osteomyelitis is rare, our patient was managed successfully by urgent wound debridement, administration of broad-spectrum antibiotics followed by VAC dressing system and skin grafting. This is only possible with a well-coordinated multi-disciplinary team consisting of a general surgeon, plastic surgeon, microbiologists and tissue viability nurses.

## Competing interests

The authors declare that they have no competing interests.

## Authors' contributions

SK Involved in the literature review, manuscript preparation and manuscript editing.

MEOD Involved in the conception of the report, literature review, manuscript preparation, manuscript editing and manuscript submission.

KK Involved in the manuscript editing and manuscript review.

GD Involved in the manuscript editing and manuscript review.

PDC Involved in the manuscript editing and manuscript review.

JL Involved in the conception of the report, manuscript editing and manuscript review.

All authors have read and approved the final manuscript.
